# Simplification to Stribild vs continuation of RTV-boosted DRV with FTC and TDF in virologically suppressed HIV adults: a STRATEGY-PI subgroup analysis

**DOI:** 10.7448/IAS.17.4.19805

**Published:** 2014-11-02

**Authors:** Jose Arribas, Giuliano Rizzardini, Keikawus Arasteh, Christine Zurawski, Craig Dietz, Dennis Pontani, Will Garner, Thai Nguyen

**Affiliations:** 1 HIV Unit, Hospital La Paz, IdiPAZ, Madrid, Spain; 2 Infectious Diseases, Luigi Sacco Hospital, Milan, Italy; 3 Infectious Diseases, EPIMED, Vivantes Auguste-Viktoria-Klinikum, Berlin, Germany; 4 Infectious Diseases, Atlanta ID Group, Atlanta, GA, USA; 5 HIV Medicine, Kansas City Free Health Clinic, Kansas City, MO, USA; 6 HIV Division, Gilead Sciences, Foster City, CA, USA

## Abstract

**Introduction:**

Simplification to Stribild (STB) was statistically superior to continuation of a ritonavir-boosted protease inhibitor (PI+RTV) with emtricitabine and tenofovir DF (FTC/TDF) at week (W) 48 in virologically suppressed HIV adults (1). We report the W48 efficacy and safety of STB versus RTV-boosted darunavir (DRV) with FTC/TDF in suppressed subjects.

**Material and Methods:**

Virologically suppressed subjects on PI+RTV with FTC/TDF regimens for ≥6 months were randomized (2:1) to switch to STB vs continue their PI regimen. Eligibility criteria included no documented resistance to FTC and TDF, no history of virologic failure and eGFR ≥70 mL/min. The primary endpoint was the proportion of subjects in the modified ITT population who maintained HIV-1 RNA <50 copies(c)/mL at W48 by FDA snapshot algorithm (12% non-inferiority margin). Subgroup analysis by PI use (DRV [173], atazanavir [174], lopinavir [72], Other PI [13]) at screening was pre-specified.

**Results:**

Four hundred twenty-nine subjects were randomized and treated (mITT set). In the DRV subgroup, 113 switched to STB; 60 continued a RTV-boosted DRV with FTC/TDF. At W48, 95% STB versus 92% DRV maintained HIV-1 RNA <50 c/mL. No emergent resistance was detected in either group. Median increases from baseline in CD4 count at week 48 (cells/µL): 28 STB versus 29 DRV (p=0.81). Discontinuations due to adverse events were 3% STB versus 2% DRV; one case of isolated decrease in eGFR in the DRV group and no cases of proximal renal tubulopathy in either group. There were statistically significant decreases in the frequency of diarrhoea reported on the HIV Symptom Index at week 4 to week 48 compared to baseline after switching to STB. There was a greater but non-progressive decrease from baseline in eGFR in the STB vs DRV group; median changes (mL/min) at week 48: −8.5 vs −0.6, consistent with the known cobicistat inhibition of renal creatinine secretion. Switch to STB was associated with a higher treatment ease (convenience, flexibility, demand, lifestyle, understanding) score (range: −15 to 15) at week 4 (median: 12 vs 9; p=0.006) and week 24 (median: 13 vs 8; p=0.001).

**Conclusions:**

In this small group of virologically suppressed subjects, simplification to STB versus continuation of a RTV-boosted DRV with FTC/TDF was safe, well-tolerated, and associated with a high rate of virologic suppression at week 48. There was more treatment ease with STB use.
